# Hydroxyethyl starch versus Ringer solution in cardiopulmonary bypass prime solutions (a randomized controlled trial)

**DOI:** 10.1186/1749-8090-3-45

**Published:** 2008-07-12

**Authors:** Osman Tiryakioğlu, Gürdeniz Yıldız, Hakan Vural, Tugrul Goncu, Ahmet Ozyazıcıoglu, Şenol Yavuz

**Affiliations:** 1 Department of Cardiovascular Surgery, Bursa Yuksek Ihtisas Education and Research Hospital, Bursa, Turkey; 2 Department of Cardiovascular Surgery, Adana Numune Education and Research Hospital, Adana, Turkey

## Abstract

**Background:**

In our study we compared the Ringer solution, which is the standard prime solution of our department, with the HES (Hydroxyethyl starch) 130-0.4 solution, which can be a potential alternative prime solution with an indispensable material for the cardio-pulmonary bypass applications.

**Methods:**

140 patients undergoing to CABG (Coronary Artery Bypass Graft surgery) were electively enrolled to the study. 1500 ml Ringer solution + 200 ml mannitol + 60 ml sodium bicarbonate + 150 U/kg heparin was used as a prime solution to start cardiopulmonary by-pass in 70 patients which was defined as group 1. On the other hand, 1500 ml HES 130 - 0.4 + 200 ml mannitol + 60 ml sodium bicarbonate + 150 U/kg heparin was used as a prime solution in 70 patients in group 2.

**Results:**

INR (International Normalized Ratio), urea levels and blood platelet counts were significantly different between the groups. INR level was higher in group 1, while blood urea and creatinine levels and platelet count were higher in group 2 at the end of the 12th and 24nd hours postoperatively (p = 0.001).

In this study, it was shown that the usage of HES 130-0.4 as a prime solution did not have negative effect on postoperative INR level, platelet count, the need for transfusion and the amount of drainage, despite the negative opinions that similar solutions caused coagulation disorders. Another interesting result of the study was that blood platelet count at 24th hour was statistically significantly higher in group 2 (p = 0.001).

**Conclusion:**

HES 130-0.4 solution is an alternative colloidal solution which can be used as the prime solution or as a mixture with the crystalloids in cardio-pulmonary bypass applications.

## Introduction

The heart-lung machine and the corresponding lines must be prepared before beginning the cardiopulmonary bypass procedure. To fill the venous reservoir and the lines with fluid and to sweep away the air particles in the lines from the system, approximately 1500–2000 ml fluid is needed. Balanced electrolyte solutions, starch solutions, gelatin or mixtures of these solutions with predetermined ratios are used [1].

Prime solutions are solutions which are used to prepare the extracorporeal perfusion line in cardiopulmonary bypass applications. It is prepared with balanced electrolyte solutions with pH and ionic distribution similar to those of the plasma, and sometimes starch solutions, which generally do not contain erythrocytes, are added. In adults, the amount of prime solution is approximately two liters. Blood is not used in prime solutions, except for those used in anemic patients or the infants. Initial experiences with prime solutions which were prepared only with blood were unsuccessful and caused homologous blood syndrome characterized with accumulation of blood in splenic bed and a shock scene [1-4].

30% reduction in the renal blood flow accompanies the renal vascular resistance during the cardiopulmonary bypass. Ischemia caused by this condition disturbs the glomerular and tubular functions. Hemodilution has considerable beneficial effects on this situation. It enhances renal blood and plasma flow, free water and creatine clearance, glomerular filtration and the urine volume. It causes significant restoration in the blood flow of renal outer cortex [4].

Disadvantage of the haemodilution is that it reduces the intravascular osmotic pressure and subsequently, interstitial edema develops. The excessive expansion of the interstitial fluid can have some drawbacks. The foremost drawbacks are the disturbance of the lymphatic drainage and the increase of the interstitial fluid volume. Consequently, malfunctioning of many organs, especially lungs, decrease in pulmonary compliance, hypoxia, peripheral edema, edema in muscles and gastrointestinal system and disturbance of the tissue oxygenation can occur [1].

This study is aimed to evaluate the effects of the excessive expansion of the interstitial fluid through administering the colloidal HES 130-0,4 instead of the classic method of crystalloid Ringer solution as the prime solution and its leading to interstitial edema and to interrogate the concepts of the increase in bleeding tendency by utilizing similar starch solutions and the unfavorable effects on renal functions.

## Methods

140 patients have been studied, who were subjected to elective CPB interventions at our Department between February and December 2006. Written informed consent for participation was obtained, and the hospital ethics committee approved the protocol.

Patients with renal insufficiency preoperatively were excluded from this study. The study of 140 subjects with no demographic differences was arranged by separating them as Group 1 consisting of 70 subjects (Ringer solution) and Group 2 consisting of 70 subjects (HES 130-0.4).

To begin the cardiopulmonary bypasses on 70 subjects of Group 1, 1500 ml Ringer solution + 200 ml mannitol + 60 ml sodium bicarbonate + 150 IU/kg heparin was used as the prime solution. On the other hand, to begin the cardiopulmonary bypasses on 70 subjects of Group 2, 1500 ml HES 130-0,4 (Voluven^®^; Fresenius Kabi Deutschland GmbH, Bad Homburg, Germany) + 200 ml mannitol + 60 ml sodium bicarbonate + 150 IU/kg heparin was used as the prime solution.

Subjects were introduced to the cardiopulmonary bypass by applying 2.2 l/min/m^2 ^total systemic blood flow at moderate hypothermia of 26–31°C. The hematocrite value of the subjects was kept at 20–25 during the process of cardiopulmonary bypass.

To ensure standardization between the groups, subjects were classified according to age, sex, cross-clamp time, cardiopulmonary bypass duration, coronary bypass number, diabetes, chronic obstructive pulmonary disease, hypertension, hypercholesterolemia, preoperative coronary lesions, executed coronary bypass number and LIMA (Left Internal Mammary Artery) usage. In the morning of the operation day, 12 derivation electrocardiograms of the subjects were executed.

### Surgical procedure

All patients underwent standard median sternotomy. Cardiopulmonary bypass was introduced by arterial cannulation from the ascending aorta and by two stage venous cannulation from the auricle of the right atrium. Cardioplegia cannula was positioned into the root of the aorta and cardioplegia was given to all the patients through antegrade way.

The prepared LIMA graft was anastomized in all patients to the descending left anterior coronary artery (LAD). Membrane oxygenator was used in all patients. Hematocrite was kept at 20%–25% throughout the CPB. Pump flow of 2–2.2 l/min/m^2 ^and nonpulsatile and median arterial pressure through the cross-clamp was kept at 50–60 mmHg. Following the cross clamp, the warming procedure was continued until the bladder temperature reached the 36°C.

At the 12th and 24th hours following the operation, hematocrite, hemoglobin, platelet counts, INR, SGOT, SGPT, urea, creatinine, sodium, potassium, chloride, and calcium levels were measured in all patients and medians in the two groups and statistical analysis were evaluated. Medians in two groups and statistical analysis of the amount of the used prime solution, the amount of the fluid added to the pump during the CPB, net balance at the pump output, postoperative drainage, the amounts of postoperative blood and FFP (fresh frozen plasma) transfusion, extubation time, the duration of intensive care and discharge time were evaluated.

At the 72nd hour postoperatively, the creatine clearances of all the subjects were obtained and their statistical analysis were evaluated.

### Statistical Method

For the analysis of the demographic, preoperative, operative, and postoperative data and the results of each group with the method of median +/- t standard deviation and with the utilization of SPSS 11.0 statistics software to get the categorical data X^2 ^(chi-square) and to get the multiple variables, the One-Way Anova and the Independent-Samples-T Test methods were utilized. Values lower than 0.05 for p value were considered as significant.

## Results

There is no significant difference between the risk factors including the demographic and preoperative specifications of both groups (Table 1, Table 2).

**Table 1:** Demographic Specifications.

	Group-1	Group-2	P value
Age	58 ± 7	56 ± 8	NS
Sex M/F	60/10	60/10	NS
Height (cm)	168 ± 8	167 ± 8	NS
Weight (kg)	78 ± 9	75 ± 11	NS
BSA	1.88 ± 1.2	1.85 ± 1.4	NS
Diabetes	20(%28.5)	22(%31.4)	NS
Hypercholesterolemia	20 (%28.5)	20 (%28.5)	NS
COPD	4(%4)	4(%4)	NS
Hypertension	30(%42.8)	30 (%42.8)	NS
Sick vessel number	3(%100)	3(%100)	NS
Graft number	3.4 ± 0.62	3.48 ± 0.58	NS
LİMA usage	70 (%100)	70 (%100)	NS

(COPD: Chronic Obstructive Pulmonary Disease. BSA: Body Surface Area. LİMA: Left internal mammarian artery. NS: Not significant (p > 0.05)).

**Table 2:** Preoperative Specifications.

	Group 1	Group 2	P value
Hematocrite(%)	42 ± 3	41 ± 4	NS
Hemoglobin(gr/dL)	14 ± 1	14 ± 1	NS
Platelet(10^3^/μl)	240 ± 80	272 ± 60	NS
INR	1.07 ± 0.12	1.04 ± 0.15	NS
SGOT(U/L)	20 ± 2	26 ± 4	NS
SGPT(U/L)	44 ± 10	43 ± 8	NS
Urea(mg/dL)	37 ± 10	38 ± 11	NS
Creatine(mg/dL)	1 ± 0.2	1.1 ± 0.1	NS
Creatinine clearance	76 ± 8	82 ± 6	NS
Na (meq/L)	139 ± 8	140 ± 4	NS
K (meq/L)	4.6 ± 0.2	4.4 ± 0.3	NS
Cl (meq/L)	101 ± 5	102 ± 4	NS
Ca (mg/dl)	8.8 ± 0.5	9.1 ± 0.1	NS

NS: Not significant (p > 0.05).

Median data and statistical analysis of the parameters which were compared at the 12th and 24th hours postoperatively are documented in Table 3 and 4.

**Table 3:** Data of postoperative 12^th ^hours.

	Group 1	Group 2	P value
Hematocrite(%)	29 ± 4	29 ± 3.5	NS
Hemoglobin(gr/dL)	10 ± 1	10 ± 1	NS
Platelet(10^3^/μl)	155 ± 25	175 ± 30	NS
INR	1.39 ± 15	1.05 ± 9	0.0001*
SGOT(U/L)	73 ± 16	85 ± 20	NS
SGPT(U/L)	40 ± 15	42 ± 18	NS
Urea(mg/dL)	30 ± 12	41 ± 15	0.0001*
Creatine(mg/dL)	1.1 ± 0.3	1.3 ± 0.3	NS
Na (meq/L)	137 ± 6	138 ± 6	NS
K (meq/L)	4.1 ± 0.2	4.1 ± 0.3	NS
Cl (meq/L)	103 ± 5	102 ± 4	NS
Ca (mg/dl)	7.4 ± 0.5	7.4 ± 0.6	NS

NS: Not significant (p > 0.05).

**Table 4:** Postoperative data at the 24^th ^hour.

	Group 1	Group 2	P value
Hematocrite(%)	28 ± 4	29 ± 3.5	NS
Hemoglobin(gr/dL)	9 ± 1.5	10 ± 1.3	NS
Platelet(10^3^/μl)	148 ± 36	189 ± 66	0.001*
INR	1.38 ± 0.19	1.12 ± 0.09	0.001*
SGOT(U/L)	71 ± 13	99 ± 17	NS
SGPT(U/L)	43 ± 11	44 ± 12	NS
Urea(mg/dL)	32 ± 10.3	46 ± 12.3	0.001*
Creatine(mg/dL)	1,1 ± 0.22	1,4 ± 0.24	0.001*
Na (meq/L)	133 ± 6	135 ± 8	NS
K (meq/L)	4 ± 0.9	4.1 ± 0.8	NS
Cl (meq/L)	100 ± 6	101 ± 6	NS
Ca (mg/dl)	7.5 ± 1.2	7.5 ± 1.3	NS

SGOT; Serum glutamic oxaloacetic transaminase, SGPT; Serum glutamic pyruvic transaminase. NS: Not significant (p > 0.05).

There were no significant difference regarding the hematocrite, platelet, SGOT, SGPT, creatine, sodium, potassium, chloride and calcium values at postoperative 24th hour between two groups. However, there is a significant difference regarding the INR, urea and creatine values at postoperative 24th hour (p = 0.001). The INR values of Group 1 were found to be higher at postoperative 24th hour. On the contrary, platelet count, serum urea and creatinine values were higher in Group 2.

Consequently, it can be suggested that the utilization of HES 130 - 0.4 as the prime solution has a negative effect on renal functions. Although the serum urea and creatine values were higher than the Ringer group, they were still within the normal range and it was demonstrated that they do not cause any serious renal damage and renal insufficiency. On the other hand, HES 130-04 solution has to be utilized very carefully on subjects with disturbed renal function and on borderline patients, taking into account the benefit/harm relationship.

Some operative and postoperative data are given in Table 5. No significant difference could be established between the postoperative blood and FFP utilization, postoperative drainage, extubation time, intensive care duration and discharge time.

**Table 5:** Operative and postoperative data.

	Group 1	Group 2	P value
ACT (A) value	128 ± 18	132 ± 16	NS
ACT (after protamine)	138 ± 24	143 ± 32	NS
Aortic cross clamp duration (min)	85 ± 11	90 ± 13	NS
Cardiopulmonary bypass duration (min)	100 ± 23	108 ± 25	NS
Prime solution amount (ml)	1500	1500	NS
Fluid added to the pump during CPB (ml)	1360 ± 360	0	0.0001*
Net balance at the pump outlet (ml)	1515 ± 270	590 ± 100	0.0001*
Postoperative blood transfusion (units)	2	2	NS
Postoperative FFP transfusion (units)	2	2	NS
Postoperative drainage (ml)	460 ± 140	430 ± 150	NS
Extubation time (hour)	10 ± 4	9 ± 4,5	NS
Intensive care stay (hour)	47 ± 12	45 ± 10	NS
Creatine clearance at postoperative 72nd hour	68 ± 15	64 ± 15	NS
Discharge time (days)	9 ± 3	8 ± 3	NS

ACT: Active coagulation time; CPB: Cardiopulmonary bypass; FFP: Fresh frozen plasma; NS: Not significant (p > 0.05).

There is a significant difference in the used prime solution volume during the CPB regarding the fluid volume added to the pump and the net balance at the pump outlet (p = 0.001).

The prime solution volume and the net balance at the pump outlet of the group of HES 130-0.4 were significantly lower. Approximately 1360 ml of fluid was added to the pump of the patients with Ringer solution group during the CPB. In the HES 130-0.4 group, there was no need for feeding any additional fluid to the pump.

Although the fluid loading and the interstitial edema were significantly lower in the HES 130-04 group, there was no statistically significant difference regarding to the extubation, intensive care stay and discharge time, which can be considered as criteria basic for the evaluation of the subjects. Nevertheless, it is evident that the medians of these intervals are shorter than that of the Ringer solution group. We thought that, by comparing groups with more subjects, a statistically significant conclusion can be reached for these parameters.

No significant difference could be established regarding the creatine clearance of both groups at the 72nd hour. Nevertheless, by scrutinizing the creatine clearance of both groups after CPB, it can be seen that there is a decrease in the median values of both groups; this decrease is more marked for the HES 130-0.4 group. By comparing the creatine clearance of both groups at the 72nd hour, it can be seen that the creatine clearance is numerically lower for the HES 130-0.4 group. As we emphasized while evaluating the former results, it can be thought that the HES 130-0.4 solution gives more adverse effect on the renal functions.

With respect to the postoperative INR, platelet count, transfusion need and drainage amount, it can be seen that there is no increase in the bleeding tendency and no disturbance of the coagulation functions with the usage of HES 130-0.4 solution as the prime solution, as it was before with similar starch solutions. Interestingly, it was found out that the platelet count at the 24th hour postoperatively was statistically significantly higher for the HES 130-0.4 group.

## Discussion

The Ringer solution which is used as the prime solution during CPB causes edema through decreasing the colloidal osmotic pressure. As a result of edema, which affects many tissues, various organ dysfunctions occur after CPB. It is still disputed whether to use crystalloid or colloidal solution as the prime solution. Many studies are both for and against one or the other [2].

HES controls the transition of the fluid into the interstitium through CPB. Laubenthal and Messmer were able to demonstrate by their study that the HES colloidal solution possesses a higher oncotic effect than albumin. Colloidal particles show their effect through the semipermeable membrane of the capillaries. This effect diminishes the transition of the fluid to the interstitium [3].

In our study, it was found out that the net balance was +1515 ml for the Ringer group and +590 ml for the HES group. This result has shown that the net balance was statistically significantly lowered down for the HES group.

Extubation time was determined as approximately 232 minutes for the Ringer group and 173 minutes for the HES group. It was determined that maximum edema formation was in the group where only Ringer was used as the prime solution. They found out that there was a significant shortening of the ventilation time in favor of the HES group [2].

In our study, no statistically significant difference could be established between the two groups regarding the extubation times, intensive care duration and discharge time.

Nevertheless, it was established that the mean values of these criteria were shorter in the HES group. By comparison of groups with bore subjects, it can be possible to establish a statistically significant result for these parameters.

The potential relationship between the intraoperative HES utilization and the postoperative bleeding is a concept which is still being discussed without any undivided opinion yet.

Complications of bleeding after open heart surgeries are often encountered. Previous studies have shown that 0.6%–15% of the patients have to be revised because of bleeding. In less severe cases, the bleeding has been treated with blood transfusions and with the methods of regulating the coagulation pattern. Clinical and economical effects of the bleeding complications after CPB are very extensive. Consequently, attempts to reduce postoperative bleeding are gaining importance. Two of the determined risk factors are age and female sex.

One of the risk factors which can be manipulated is the utilization of perioperative HES [3-7].

In the patients of HES 130-0.4, the increase in the von Willebrand factor were more pronounced while the blood loss and the usage of erythrocyte concentrate were less [8].

In our study, the postoperative drainage volume was approximately 460 ml for the group of Ringer solution as the prime solution and approximately 430 ml for the group of HES 130-0.4; no statistical difference between the two groups could be established.

The increase of renal vascular resistance during the CPB is accompanied by the 30% decrease of the renal blood flow. The resulting ischemia disturbs the glomerular and tubular functions.

Besides, it is well known that diuretics, aprotinine, preoperative opaque material intake and many medications used during the CPB have unfavorable effects on the renal functions. When haemodilution is not used, CPB will decrease the free water and creatinine clearance in conjunction with urine volume. Haemodilution has considerable benefits on this subject. It increases the renal blood and plasma flow, free water, creatinine clearance, glomerular filtration rate and urine volume; it particularly causes considerable improvement on the blood flow of renal outer cortex [4].

Some case reports and clinical information claim that the utilization of hydroxyethyl starch can destroy the renal functions, but this assertion was investigated only in a small number of studies and the results are controversial.

In their study of patients who underwent CPB, Winkelmayer et al. have determined that HES utilization was related with the independent decrease in the glomerular filtration velocity at the 3rd and 5th days after surgery. They pointed out that the intraoperative HES utilization in patients of coronary artery bypass surgery will cause a slight disturbance of renal functions [9].

In another study of Boldt et al. of patients older than 70 years, gelatin was compared with HES 130-0.4 and they could not establish any difference between the two volume replacement applications regarding the renal integrity [10].

In the study of Maha et al. for the determination of optimal amount of HES to use as the prime solution in CPB patients, mean urine volume was 2003 ml in the Ringer group and 1710 ml in the HES group. Although there was a decrease in the HES group, there was no statistically significant difference between the two groups [2].

In our study, urea values at the 12th hour postoperatively were 30 mg/dl for the Ringer group and 41 mg/dl for the HES group. These results are statistically significant and show that the urea values are higher in the HES group. Creatine values at the 12th hour postoperatively were 1.1 mg/dl for the Ringer and 1.3 mg/dl for the HES group. There is no statistically significant difference between the two groups.

Urea value at the 24th hour was mean 32 ± 10.3 mg/dl for the Ringer group and mean 46 ± 12.3 mg/dl for the HES group. Creatine value was 1.1 mg/dl for the Ringer group and 1.4 mg/dl for the HES group. These values show the statistically significant difference between the two groups.

Creatinine clearance which was investigated at the postoperative 72nd hour was mean 68 ± 14 in the Ringer group and 64 ± 14 in the HES group. By comparison, it comes out that there is no statistical difference between the two groups.

Consequently, it can be suggested that the utilization of HES 130-0.4 as the prime solution has unfavorable effects on the renal functions. Although the serum urea and creatine values were higher than those of the Ringer group, these values were within the normal range and it was seen that they did not cause any serious renal damage and renal insufficiency.

In conclusion, HES 130-0.4 solution is an alternative colloidal solution which can be used as the prime solution in cardio-pulmonary bypass applications.

## Authors' contributions

OT conceived of the study, design of the study and performed the statistical analysis. GY conceived of the study, and participated in its design and coordination. HV participated in the study design and coordination. TG design of the study and performed the statistical analysis. AO participated in the study design and coordination. ŞY participated in the study design and coordination. All authors read and approved the final manuscript.
